# The Exceptions and the Rules in Global Musical Diversity

**DOI:** 10.5334/joc.312

**Published:** 2023-08-18

**Authors:** Sam Passmore, Patrick E. Savage

**Affiliations:** 1 Faculty of Environment and Information Studies, Keio University, Fujisawa, Japan; 2 Evolution of Cultural Diversity Initiative, Australia National University, Australian Capital Territory, Australia; 3 School of Psychology, University of Auckland, New Zealand

**Keywords:** Music, Diversity, Cross-Cultural

## Abstract

Global music diversity is a popular topic for both scientific and humanities researchers, but often for different reasons. Scientific research typically focuses on the generalities through measurement and statistics, while humanists typically emphasize exceptions using qualitative approaches. But these two approaches need not be mutually exclusive. Using a quantitative approach to identify musical outliers and a qualitative discussion of the most unusual songs, we can combine scientific and humanities approaches to unite knowledge on musical diversity. Objectively defining unusual music is a delicate task, having historically been subject to Eurocentric approaches. Using the Global Jukebox, a dataset containing almost 6,000 songs from over 1,000 societies coded on 37 “Cantometric” variables of musical style, we designate the unusualness of a song as the frequency of its coded variables relative to their regional frequency. Using quantitative metrics to identify outliers in musical diversity, we present a qualitative discussion of some of the most unusual individual songs (from a Panpipe ensemble from Kursk, Russia), and a comparison of unusual repertoires from Malay, Kel Aïr, and Moroccan Berber musical cultures. We also ask whether unusual music is the result of unusual social organisation or isolation from other groups. There is weak evidence that the unusualness of music is predicted by kinship organisation and cultural isolation, but these predictors are heavily outweighed by the finding that unusual songs are best predicted by knowing the society they come from – evidence that quantitatively supports the existence of musical style.

Music is a universal cultural phenomenon that both captivates and bonds humans across the globe (Brown & Jordania, 2013; Mehr et al., 2019; Savage et al., 2021; Tomlinson, 2015). The function of music, whether that be for soothing or dancing, has been shown to constrain diversity between societies (Jacoby et al., Accepted; Jacoby & McDermott, 2017; Mehr et al., 2018; Yurdum et al., 2022), but within societies, music plays an important role in defining cultural identity (Margulis et al., 2022; Tomlinson, 2015; Trehub et al., 2015). Although there has been progress in identifying the cross-cultural *similarities* in musical production and perception (Bainbridge et al., 2021; Hilton et al., 2022; Jacoby et al., Accepted; Mehr et al., 2019; Savage et al., 2015), this research leaves a founding question of ethnomusicology largely unanswered: what makes music *different* (Nettl, 2015)? Research focusing on differences has shown cross-cultural variability in tonal preferences (McDermott et al., 2016) and emotional responses to music (Smit et al., 2022) which highlight the importance of understanding cultural influence on musical diversity. These studies show cultural variability through pairwise comparison of Western and non-western societies. Here, we use quantitative metrics in a global sample of songs and societies to identify musical outliers, and qualitatively discuss their contexts to identify what features might explain how cultural contexts change music.

Quantifying musical differences between societies is fraught with empirical and epistemological difficulties (Jacoby et al., 2020). Mounting evidence suggests that musical diversity is higher within societies than between them (Daikoku et al., 2020; Mehr et al., 2019; Rzeszutek et al., 2012). The high within-society variance might be attributed to universal constraints imposed by the song genre. For example, Lullabies tend to have gentle and slow tempos, and dance songs tend to have fast tempos across societies means we will see a high within-society variance in tempos due to the coexistence of these two genres within each society (Mehr et al. 2019). As a result of how music interacts with parts of neurological circuitry, we find acoustic regularities between societies in the performance of lullabies that we can reasonably expect to be found universally (Yurdum et al., In press). These findings suggest that to understand how music varies between societies, we must be conscious of how frequently different musical features occur universally and ensure that frequency is controlled for in any cross-cultural comparison (see Savage et al., 2015 for some examples).

Another central challenge is creating a cross-culturally valid system for defining and/or comparing music (Savage, 2022). Quantifying cross-cultural musical diversity was the focus of Alan Lomax’s Cantometrics and Global Jukebox projects which collated corpora of data covering performing arts and culture from around the world (Wood et al., 2022; Figure 1). The Cantometrics sub-project presented a cross-cultural framework for the comparative study of song (Lomax, 1976; Savage, 2018). Cantometrics quantifies 5,776 traditional songs from 1,026 societies (5,484 songs from 993 societies excluding missing data) on 37 features designed to capture cross-cultural features of music and avoid the biases imposed by Western staff notation (Wood et al., 2022). Although the sampling and coding of Cantometrics have had its critics (see Savage, 2018 for a review), it remains the largest digitised and quantified source of cross-cultural data on musical diversity, and has undergone significant data cleaning and validation before the recent re-release (Wood et al., 2022). In this paper, we follow recent work in linguistics to examine the unexpected variation in global musical diversity, which we call unusualness (Skirgård et al., 2023). Here, unusualness is a measure of relative feature frequency for each song which will allow us to analyse cross-cultural variation in musical performance and captures musical diversity in a framework designed for cross-cultural comparison.

**Figure 1:**
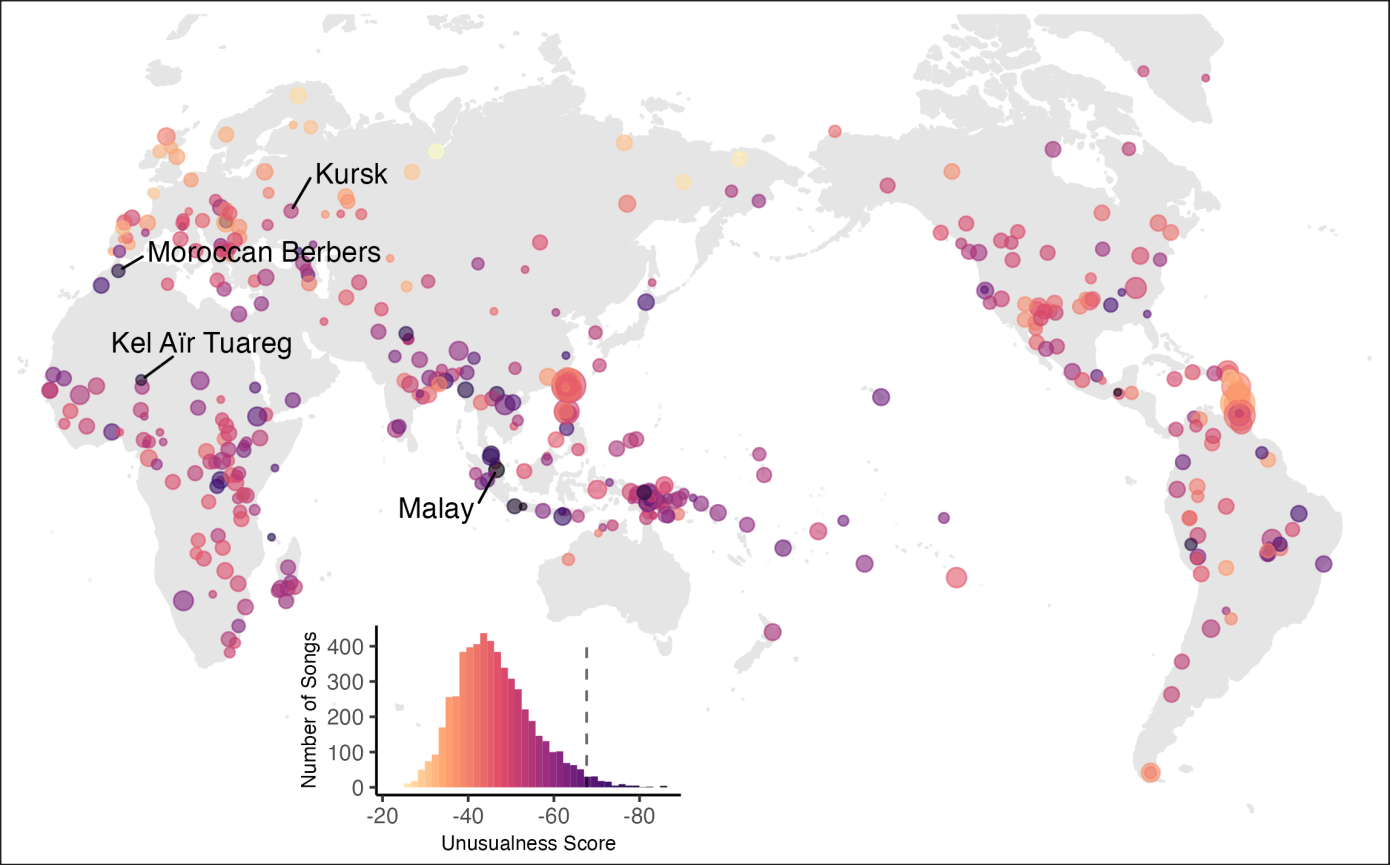
Map of 993 societies represented in Cantometrics, sized by the number of songs (a total of 5,484 songs, the maximum number within one society is 72, and the minimum is 1). The inset histogram shows the global distribution of song unusualness, with a dotted line showing the top 3% of unusual songs.

Unusualness as a concept can be defined in many, often Eurocentric ways but has rarely been measured quantitatively (Savage, 2022). One study by Panteli, Benetos and Dixon (2017) used Music Information Retrieval (MIR) to quantitatively identify musical outliers in a global sample, but because they did not have any ground-truth human-annotated data on musical features their results are difficult to interpret from a musicological perspective (Ozaki et al., 2021). Research on the interpretability of MIR features found no correlation between Panteli et al.’s MIR features and similarity ratings derived by humans for a global sample of songs, suggesting these dimensions do not track perceptual proxies of musical interpretation (Daikoku et al. 2022).

Utilising the cross-cultural framework and human “Cantometric” annotated data for many songs allows us to calculate unusualness from the relative regional frequency of their annotations, also known as the log-likelihood (Figure 2). There are 6 repeating steps to calculate unusualness. First, identify a region. In Figure 2, this is South America. Second identify a particular society, Canela. The third step is to calculate how often each state occurs for each feature in all South American songs that are not performed by Canlea performers. This offers a measure of how common (or unusual) each state within each feature is. A state is a particular code within a Cantometric feature. For example, the Cantometric feature for tempo (24) has six states: very slow, quite slow, slow, moderate, fast, and very fast. The fourth step is to calculate unusualness, by using the state probability relating to the coding for each Canelo song. See the figure caption for a walkthrough of one example. The fifth step is to repeat steps two to four for all other South American societies, and the sixth and final step is to repeat all steps for the next region until all regions have been analysed.

**Figure 2:**
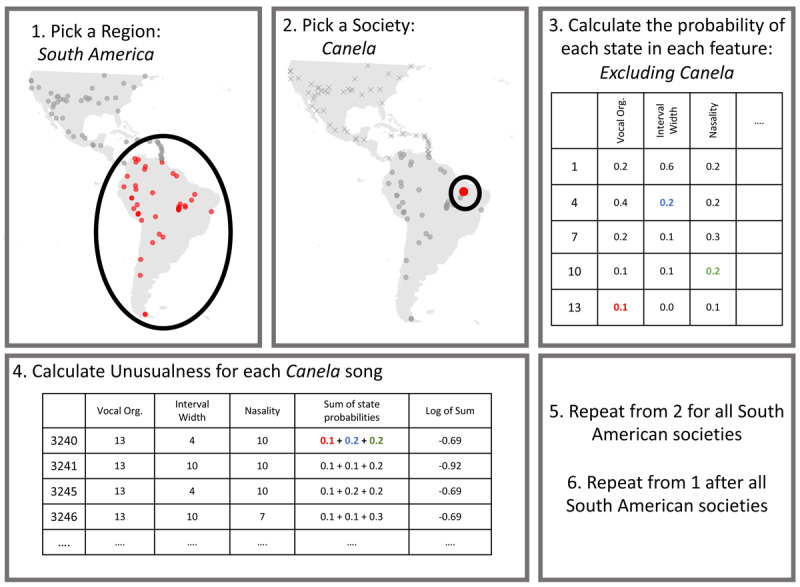
The six steps for calculating Unusualness for any particular song are as follows. 1) Identify a region of interest (South America). 2) Identify a society of interest (Canela). 3) For all songs, excluding songs by Canela performers, calculate the probability of each state within each feature (Note the values in the figure are for illustrative purposes only). 4) For each Canelo song, take the state probability for the value coded within that song and sum it with all other state probabilities. For example: in song 3240, the Vocal Organisation variable is coded as 13 (Polyphony, red). This occurs in 10% of all South American songs, or 0.1. For interval width, song 3240 is coded as a 4 (Narrow intervals), this occurs in 0.2 of all other South American songs (blue). Nasality is coded as 10 (Slight nasalization), which occurs in 0.1 of all other South American songs. We sum these state probabilities together and take the log of the summed value to calculate unusualness (the log-likelihood). 5) Repeat from step 2 for all societies in South America. 6) Repeat from step 1 once all South American societies have been calculated.

Conceptually, our measure of unusualness indicates how different a song is from other songs recorded in the same region (excluding songs from the same society). The frequency-based measurement of unusualness offers a control for contextual regularities since features that occur near-universally across societies occur often and are not unusual. By calculating unusualness within regions, we also control for some common critiques of Cantometrics data (cf. Savage, 2018): firstly, it means we allow for unbalanced sampling by regions. Secondly, it identifies songs that are unusual within a geographic region where we might expect songs to be more similar.

We use the quantitative measure of unusualness to identify the most unusual songs and societies in our global sample. The outliers provide the basis for a qualitative discussion of the context and environment of these outliers, and why they might have become so unusual. Our discussion identifies three central theories of cultural change: cultural isolation, change in social structure, and musical style. These three theories suggest systematic reasons for unusualness. We test the relative importance of these theories in a Bayesian multi-level regression model.

## The peculiarity of Panpipes in global traditions

The two most unusual songs in the dataset were performed by a panpipe ensemble of four women in Kursk, Russia in 1991 (song id: 30076 & 30077^1^). These songs contain a combination of interlocking panpipe instruments and yodelled singing. The combination of singing with panpipe music is a particularly rare phenomenon in the Cantometrics sample. The use of panpipes is only found in a few regions in Russia: Briansk, Kaluga, and South Kursk provinces (Velitchkina, 1996). However, panpipe traditions are common throughout the world and are known to have existed in nearby Serbia, Romania, Komi Republic (North Russia, Ugro-Finnish population) and Lithuania, which are all thought to stem from the same original tradition (Žarskienė, 2003). Panpipes music had been described in Kursk society as early as 1831, despite scarce written records. The geographic spread of the tradition, and the linguistic diversity of instrument names, have led scholars to conclude it is likely an ancient tradition (Velitchkina, 1996). Panpipe playing is described as historically common, often accompanied by many other instruments (flute, fiddles, and more recently accordion), and used to accompany dances on major religious holidays. In formal settings, the music was only performed by men, but on less formal occasions women would also play. Ethnographic research in the 1990s suggests the tradition is no longer thriving, maintained by a small group of elderly women who only perform on formal occasions. Outside of the stage, the practice has been largely discontinued (Velitchkina, 1996).

The Kursk panpipe performances have been previously identified as songs whose geographical uniqueness is challenging to explain (Grauer, 2006). At first listen, the Kursk songs sound like misclassified African songs, comparable to the Luba-Lulua Dance song recorded in the Democratic Republic of the Congo.

Similar conclusions have been made by previous researchers (Grauer, 2006; Žarskienė, 2003). The identification of long-distance similarity raises the question of musical borrowings. Songs being passed between (e.g.) the Luba-Lulua and Kursk societies is unlikely in this instance for two reasons. First, migration between Africa and Russia has been historically and remains low. Global migration trends between 1846 and 1940 show that, in general, very few people moved into Russia. What migration occurred was further reduced after the World Wars as rigid passport controls came into place across Europe. Secondly, the sampling focus of the Global Jukebox was to record traditional folk songs, meaning any songs that were known to have been imported are not included. Others have noted the Kursk songs show a striking similarity to ‘Are’Are panpipe music found in the Solomon Islands (see song IDs 4246 & 9184 for examples of ‘Are’Are panpipe music) (Grauer, 2006) or Kuna panpipe music in Latin America (Žarskienė, 2003) (see song ID 9179 for an example of Kuna panpipe music).

The unusualness of the Kursk tradition in Russia, but its similarity to distant traditions highlights a constant discussion within ethnomusicology: can musical similarity tell us about ancient human movements (Callaway, 2007; Grauer, 2006; Savage & Brown, 2013, Passmore et al. 2023)? The Kursk panpipe songs stand out against the broader Russian and European traditions, but as discussed, show an uncanny resemblance to very distant traditions. Previous ethnomusicologists working with Cantometrics point to the similarity of Kursk performances to the Luba-Luba, the ‘Are’Are, and others and posited that the similarity is evidence for the preservation of a musical style that moved alongside ancient human migrations (Grauer, 2006). This claim was given some support when a global and cross-cultural comparison of panpipes construction showed that although the instruments are constructed in distinct cultural contexts, there are unexplained similarities between instruments in distinct and distant cultural regions (Aguirre-Fernández et al., 2020; Padilla-Iglesias et al., 2022). Recent work within sub-Saharan Africa has shown similarity in musical instruments, including panpipes, correlates with an ancient genetic connection between hunter-gatherer groups (Padilla Iglesias et al., 2022). Our results also show that panpipe traditions persist, despite a contrastive local tradition. This evidence presents the possibility that panpipe traditions could have a long history in human culture.

Global comparisons, archaeological evidence, and ethnographic descriptions of panpipes are pointing to long-distant diffusion events of musical traditions (Aguirre-Fernández et al., 2020). However, in the specific case of panpipes, we have not had a sufficient investigation into the possibility of convergent evolution, an important alternative hypothesis (Stock, 2006). The convergent evolution hypothesis suggests that panpipes and panpipe music are strongly constrained by ecological, physiological, and cognitive conditions. Simply, a convergent evolution hypothesis would mean the similarities in panpipe songs are not a result of tradition, but due to constraint (Scott-Phillips et al., 2021). On balance, there is much to discover on the relationship between music and its connection to human prehistory. We do not have any conclusive evidence on which of these positions is more likely to be correct. The takeaway from this section is how an exploration of outliers can quickly identify areas of musical diversity in need of further data, debate, and exploration. This case study is a good example of how a diversity-first model of musicology can identify regions of music that need to be further investigated before we can draw any conclusions. If we had a more comprehensive model of panpipe music and panpipe construction, we could draw more definitive conclusions.

## Comparative analysis of musical styles

**Figure 3:**
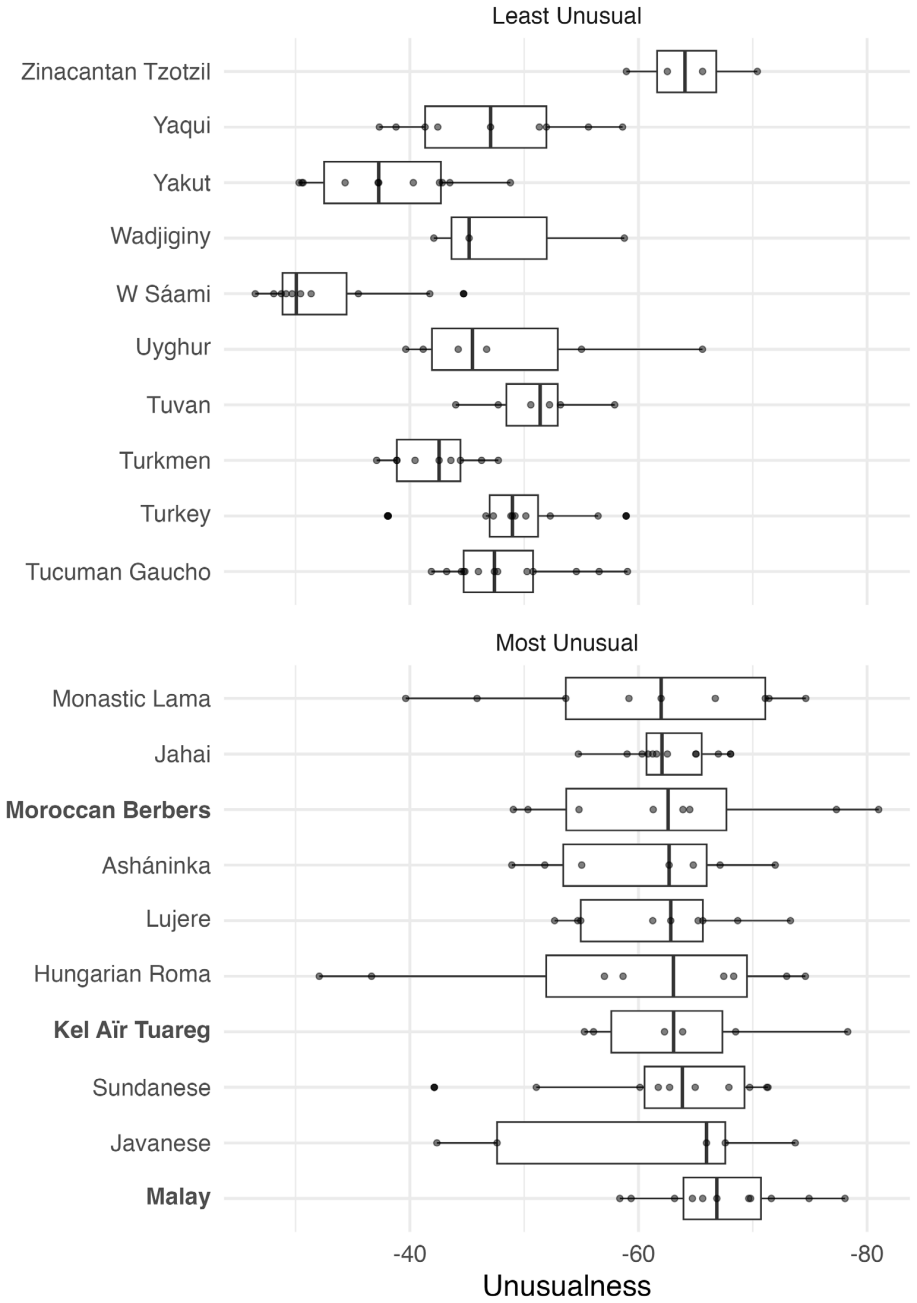
Boxplots showing the spread of unusualness within the top and bottom 10 unusual societies (from societies represented by 5 songs or more).

Although the Kursk panpipe songs are not typical of the broader Kursk repertoire, in general, we find that most songs within a society show similar levels of unusualness. We argue the consistency of unusualness within a society is indicative of a musical style. Musical style is when societies have a penchant for particular combinations of features across most songs in their repertoire. When these preferred features are unusual, then each song will be similarly unusual, which in turn makes the society unusual on aggregate. Figure 3 shows the range of unusualness for the ten most and ten least unusual societies (from societies represented by five or more songs). Although within-society diversity in unusualness is high, societies certainly sit on ends of the unusualness scale.

The society with the most unusual style is Malay performers (Figure 3). Traditional Malay music is the most unusual and stands in stark contrast to other sampled musical styles in the Southeast Asia region. The most unusual song in the Malay recordings is the religious remembrance song *Dzikhir Pahang*, and the most typical song is the magico-medical ceremonial song, *Main Putri*. The features that make Malay music unusual in the South East Asian context are the predominance of precise enunciation, narrow vocals, and extreme embellishment. In general, the Cantometric coding describes Malay music as containing little repetition, having an undulating melody, with narrow or diatonic intervals, no polyphony, and perhaps most notably containing extremely nasal vocals. These aspects are perhaps best exemplified by a song called *Wayang Kulit Siam*; a song performed alongside a shadow puppet show. This makes Malay music, and Southeast Asian music more generally, an interesting case for studying the development and change of regional diversity in music. What conditions caused Malay music to diverge from its neighbours? Does Malay society contain social or environmental conditions different to its neighbours? Or is the difference a function of different cultural lineages in Southeast Asia (i.e. Austronesian traditions compared to Austro-Asiatic or Tai-Kadai traditions)?

Within the most unusual societies are two groups of relatively close cultural heritage, the Kel Aïr (number of songs = 6) and Moroccan Berber (n = 8). The classification of North African groups as “African” within the Global Jukebox may exaggerate the unusualness of the Kel Aïr and Moroccan Berber music, who are often grouped instead with the Middle East instead of Sub-Saharan Africa (e.g., Nettl et al., 1998–2002; Savage et al., 2015). Both the Kel Aïr and Moroccan Berber societies are ethnolinguistic groups speaking languages descending from the Berber clade of the Afro-Asiatic languages family. Glottochronology suggests Moroccan Berber and Zenaga (a sister language to the Kel Aïr’s language of Tayart Tamajeq) both derive from Proto-Berber, which arose around 1,800 years ago, although some researchers claim a much earlier date (Kossmann, 2020). Despite around 72 generations of separation (at 25-year generations), the small samples of Moroccan Berber and Kel Aïr music present similar musical styles. From the point of Cantometric codings, Kel Aïr and Moroccan Berber music are indistinguishable. The similarity in musical style provides an interesting case study for why two societies may have similar musical preferences.

Musical style is often argued to reflect a combination of inherited tradition and response to the social environment (Lomax, 1980). The similarity of musical style as a result of inheritance can be described as analogous to an evolutionary process: musical traditions are passed between generations with minor changes. As changes accumulate, musical styles diverge, through a combination of random drift and directional selection (Savage et al. 2022). Social environment, on the other hand, was proposed by Lomax to regulate the interaction between individuals. Lomax viewed music as a tool to reinforce the relationships imposed by social organisation. For example: “the cohesiveness of singing groups reflects and reinforces the level of social solidarity in other aspects of social organization” (Lomax, 1989, pp. 231–233; Savage, 2018). From this hypothesis, we can predict that changes in the social organisation will change the nature of relationships between individuals in a society and therefore will change how these relationships are reinforced through music.

Despite a relatively close historical relationship, the Kel Aïr and Moroccan Berber occupy considerably different cultural niches. Different cultural niches, but similar musical styles point to the maintenance of musical tradition despite significant social change. The Kel Aïr are a nomadic Camel-riding group, inhabiting both the Aïr mountains and plains in Northern Niger with hierarchical and strong patrilineal social organisation (Card-Wendt, 2010). The Kel Aïr are often grouped into a larger group of nomadic societies and languages, classified as the Tuareg family. Ethnographic description of the broader Tuareg musical traditions tells us that the arts are highly regarded amongst the Tuareg, playing a central role in ceremonial and political life. However musical professionalism is limited to an artisanal caste and is not practised amongst the noble elites.

The Moroccan Berbers are also part of the Berber clade of the Afro-Asiatic language family but live across the Sahara in the High Atlas Mountains on the border of Morocco. Compared to the Kel Aïr, the Moroccan Berber have a more sedentary existence, living in permanent villages, and producing food via subsistence agriculture. The harshness of conditions and scarcity of resources in the Atlas mountains mean many villages have significant migration to urban centres, where most men move for work, and to relieve resource pressure on the village’s small holdings (Schuyler, 1979). Moroccan Berber music has two traditional domains, music performed within villages by the inhabitants (*ahwash*) and music performed by travelling professionals (*rwai)*. Although the professionalisation of *rwai* performance means the musicians use different instruments and perform newer songs, the *ahwash* and *rwai* music share the same melodic, poetic and musical style (Schuyler, 1979). These two traditions differ in social function, however. *Awash* requires large numbers of people and is performed at large celebrations, bringing together the members of a village for a performance. *Rwai* performers on the other hand travel between villages, making their living from performing. Oftentimes *Rwai* performers convey news from outside the village, acting like travelling journalists. Amongst both musical traditions, musical individualism is only tolerated in particularly talented or powerful individuals (Schuyler, 1979). Soloists that show enthusiasm beyond their station are often barbed for their ostentatiousness, suggesting that the Moroccan Berber will enforce tradition over individuality through social means, most of the time (Lortat-Jacob, 1979).

We noted earlier that a founding hypothesis of Cantometrics was that musical style reflected the social dynamics of societies (Lomax, 1980). In this pairwise comparison of fourteen songs (six Kel Aïr songs, and eight Moroccan Berber), we observe the continuity of musical style, despite changes in both subsistence and mobility. We might then conclude that changes in subsistence and mobility have little impact on changes in musical style. However, an important connecting factor between these societies is the connection to Islam, which is likely to have arisen between the 8th to 12th centuries (Schuyler, 1979). A common religious affiliation may have encouraged either the maintenance or convergence of musical styles leading to their contemporary similarity. Further research would need to explore which is more likely.

Although Cantometric research mentions religion as a potential motivator for determining musical style (Lomax, 1968), the influence of global religion on all forms of music is not widely explored in comparative quantitative analysis. Similarly, religious songs from Islam or other global religions are not heavily sampled in the Global Jukebox, which aims to record traditional songs, and not imported musical traditions (although the phenomenon of “invented traditions” (Hobsbawm & Ranger, 1983) renders this distinction impossible to strictly define or sample in practice). Islamic religious institutions may influence the structure of relationships within societies, which might lead to greater similarity of musical styles. The relationship of religion to social organisation has been posited to have far-reaching influence throughout the world, including via religious rituals and their invariable musical accompaniment (Atkinson & Whitehouse, 2011; Henrich, 2020; Sheehan et al., 2022; Turchin et al., 2022). In our comparative case study, change in subsistence and mobility did not substantially affect musical style, but more comprehensive comparative analyses would be required to make any strong conclusions about general relationships between music, religion, and other aspects of culture.

## The unusualness of a song is constrained by cultural styles

We used case studies to draw some conclusions on how musical style might result from unusual cultural ecologies or the influence of social organisation. Are there generalisable conclusions that we can draw from these case studies? That is, are there systematic reasons that a society’s style becomes atypical?

**Figure 4:**
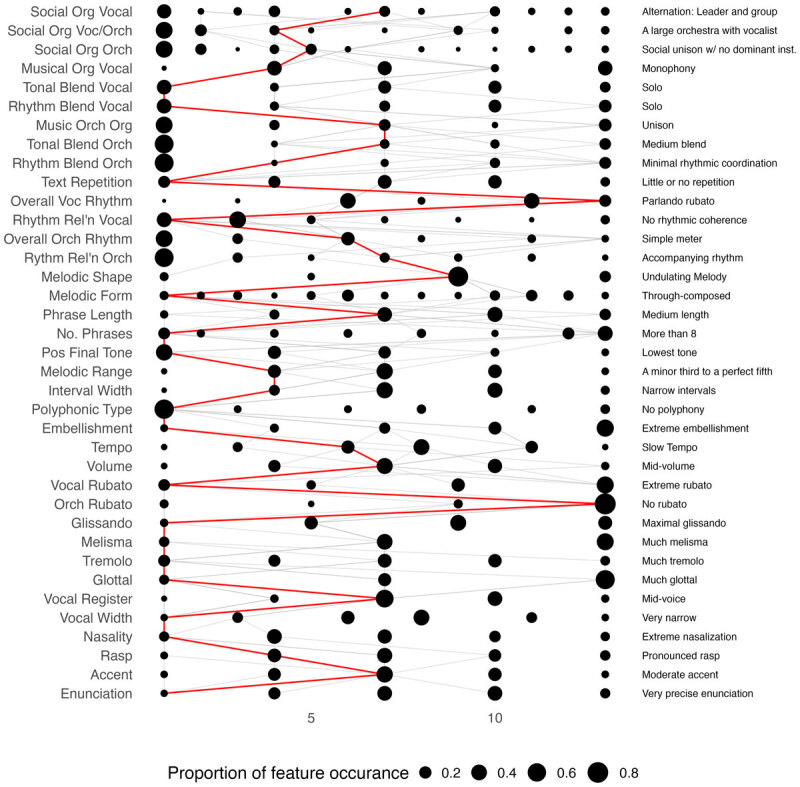
This figure shows a modified profile plot of all Malay songs (grey) and the modal profile of Malay songs (red). There are 11 songs in total. The left-hand side names each feature of Cantometrics. The right-hand side offers a written description of the modal state for each feature within Malay songs. Within each row shows a point for each possible coding of each feature, transformed to a 13-point scale. Each circle is sized by the proportion of which that coding is used in Southeast Asia (larger is more common).

Two complementary hypotheses on what might cause musical extremes arose during our qualitative discussion above. Hypothesis 1) unusualness in social organisation creates unusual music (as described in the comparison of Moroccan Berbers and Kel Aïr) and, hypothesis 2) frequency of inter-group contact encourages musical unusualness. We can infer a third hypothesis from the distribution of unusual music, seen in Figure 4, which is that unusual songs tend to come from societies with other unusual songs. The top 3% most unusual songs (N = 94), come from only 56 societies (approximately two songs per society on average), suggesting that some societies disproportionately account for unusual songs in the database.

Identifying the relationship between musical style and social structure was a primary goal of Cantometrics. Specifically, do musical styles reinforce the role of hierarchy within a society, or equally, can they encourage egalitarianism? Lomax presented his hypotheses as specific relationships. For example, societies with regular rhythm prioritise discipline, and societies that emphasise loudness place more value on military glory (Lomax, 1989; Savage, 2018). These theories emanate from the central thesis that musical performance regulated the interaction between individuals and reinforced the structure of those relationships imposed by the social organisation. We infer here that if there is a generalisable relationship between social organisation and musical performance, then we should also expect societies that are organised in unusual ways also produce unusual music. This hypothesis would then propose that an unusual social organisation will produce unusual music (hypothesis 1).

We make three predictions on the impact of inter-group contact on the unusualness of musical style, which creates three sub-hypotheses of hypothesis 2: frequency of contact (hypothesis 2a), geographic isolation (hypothesis 2b), and cultural isolation (hypothesis 2c).

Hypothesis 2a relies on the idea that music strengthens and/or signals group affiliation and unity (Mehr et al., 2021; Savage et al., 2021). If societies are in frequent contact and music is used to signal group identity, then their musical styles will need to be distinct from each other. If musical styles are not sufficiently distinguished between societies, music could not act as a group marker. As the number of societies that are in contact with each other increases, so too does the pressure to maintain a distinct musical style. With increased inter-group contact, the design space in which musical style exists becomes more crowded (Dennett, 1995), and therefore the likelihood that unusual styles arise also increases. Hypothesis 2a predicts a positive correlation between the number of close contacts and unusualness in musical style.

Hypothesis 2b, the geographic isolation of societies, predicts a negative correlation between the frequency of inter-group contact and unusual musical style, the opposite prediction from hypothesis 2a. As societies become more isolated, their musical traditions can accumulate differences that are not adopted elsewhere, making their traditions more unusual in the region.

Hypothesis 2c considers isolation in terms of cultural history, rather than the geographic isolation used in the previous two hypotheses. Cultural isolation, concerning independent histories, is a common linguistic hypothesis. Cultural Isolation suggests that languages diverge and accumulate differences over time, much like the evolution of species. Using phylogenetic techniques, models of language histories reveal how long ago languages had common ancestors (Hua et al., 2019). If we, as others have before, consider linguistic distance as a proxy for cultural distance, we might predict that increased cultural isolation allows for the accumulation of more musical innovations and the creation of more unusual music. Hypothesis 2c predicts a positive correlation between cultural distance and musical unusualness.

Finally, hypothesis 3 predicts that unusual songs will be well predicted by the unusualness of other songs within a society. This hypothesis suggests that there is an underlying constraint of musical style that restricts the make-up of songs within a society. Mathematically, the calculation of unusualness for a particular song excludes the influence of other songs from that society. This allows us to infer that the similarity in unusualness between songs from the same society is a result of them coming from the same source, rather than the relationship being a statistical artefact. We posit that if such a relationship is found it is the result of social norms producing songs with a proclivity for particular musical features, which might be indicative of musical style.

To calculate unusual social structures, we apply the same calculation that we used for song unusualness to social data from the Ethnographic Atlas, stored within the D-PLACE database (Kirby et al., 2016; Murdock, 1967). Here, societies only have one value per region, as opposed to the multiple recordings we observe in music. We calculate two different measures of societal unusualness, based on the themes of variables within the Ethnographic atlas, and have acceptable levels of data coverage: kinship organisation, and economic systems (hypothesis 1). We additionally measure general unusualness which is an aggregate measure of both kinship and economic variables. Kinship variables measure structural components of family life: marriage, family construction, community organisation, and residence patterns. Economic systems surround the method and types of food production, as well as divisions of labour, and the patterns of inheritance. To approximate the frequency of contact between societies (hypothesis 2a and hypothesis 2b), we count the number of societies within a geographic distance. We have no prior prediction of what geographic distance would be appropriate, so we test three distances of a 1,000 km, 500 km, and 250 km radius. The distance most predictive of unusualness was 500 km, identified through a bi-variate LOO (leave-one-out) model comparison. We use the 500 km radius variable in all future models. Hypothesis 2c regards cultural isolation. We measure cultural isolation by identifying the distance to the nearest phylogenetic neighbour from within an established global language tree (Bouckaert et al., 2022) (See data availability statements below for links to data/code). Hypothesis 3, the influence of musical style, is calculated for each song as the average unusualness of all other songs recorded for that society.

In total, we have six variables modelling two hypotheses to predict unusualness. Hypothesis 1 suggests that unusual social organisation will lead to unusual musical style. Unusual social organisation is measured across two dimensions of social organisation: kinship organisation, and economic systems. We also test a third measure of unusual social organisation that aggregates variables from both kinship and economics. Hypothesis 2 suggests that group contact will influence musical unusualness. Group contact is approximated through the number of geographic neighbours within 500 km. Hypothesis 2a predicts a positive correlation between the number of geographic neighbours and unusualness. Hypothesis 2b predicts a negative correlation. Hypothesis 2c is tested using linguistic phylogenetic distance, as a proxy for cultural isolation. Finally, we use the average unusualness score of all other songs from a society to test hypothesis 3 All six variables are inputted into a Bayesian multi-level regression model, implemented in R using the Bayesian modelling package, BRMS (Bürkner, 2017).

**Figure 5:**
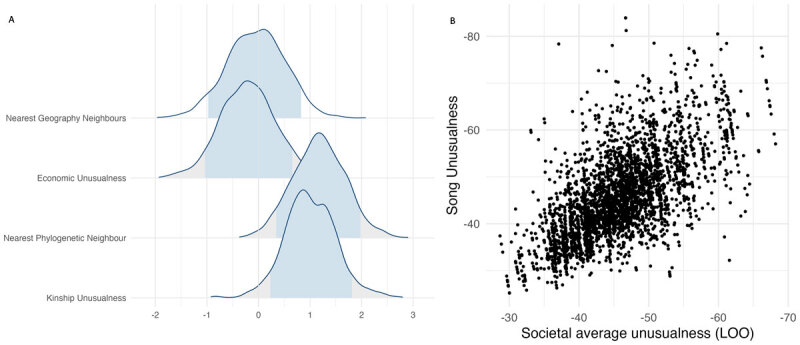
**A)** Posterior distributions of the regression coefficient estimates for the four predictor variables. Blue fill indicates 89% confidence intervals. Only the Nearest phylogenetic neighbour and Kinship unusualness show 89% confidence intervals not containing zero. **B)** Scatter plot between the unusualness score for a particular song, and the average unusualness score for all other songs within a society (Leave One Out). Scales are reversed so that further to the right of the x-axis, or higher up the y-axis is more unusual. Quantitatively smaller values are more unusual.

We first build a model containing all variables for hypotheses 1 and 2, with a random effect for society. We find weak statistical evidence for the nearest phylogenetic neighbour and kinship unusualness predicting unusualness in music since the 89% confidence intervals for these variables do not contain zero (McElreath, 2020). However, the model including these variables has a marginal R2 of less than 5%, which questions the practical significance kinship and cultural isolation have on unusualness (Figure 5a). We conclude that, although there is weak statistical support, we have no evidence that these predictors can explain musical unusualness. However, the conditional R2 for this model was close to 45%, suggesting that structuring the data to reflect societies has a considerable influence on unusualness diversity.

We replace the random effect for society with a variable showing the average unusualness for all other songs within a society (Figure 5b). The marginal R2 for this model is 45.5%. Comparing our two models, we can infer that almost 40% of the variance in unusualness is explained by knowing which society a song came from. We conclude that knowing the origin of a song (i.e. which society performed it) has both statistical and practical significance, and provides evidence for the hypothesis that musical style constrains the features of songs.

## Discussion

The quantitative expression of unusualness has helped us identify songs and musical styles that stand out from the surrounding musical traditions. We present two approaches to exploring what this quantitative measure tells us about musical diversity. Qualitative descriptions of Kursk panpipe songs, the most unusual songs in the sample, highlight the peculiarity of panpipes in human history. Panpipes have been touted as a link to historical connections between societies, but also the result of convergent evolution. Recent work on hunter-gatherer societies within Africa provides some evidence for music as a signal of ancient connectivity (Padilla-Iglesias et al., 2022), but whether this relationship applies to inter-continental ties is still a contentious conclusion (Aguirre-Fernández et al., 2020).

Our qualitative comparison of the Kel Aïr and Moroccan Berber musical styles provides a pairwise comparison of two societies that have similar and unusual styles, but decidedly different social structures. The comparison of linguistically similar and socially distinct societies highlights the contrasting nature of Lomax’s early hypotheses on musical diversity. On the one hand, Lomax proposes that musical style is constrained by social organisation, and on the other suggests that musical traits can inform us about our ancestral connections. Both of these hypotheses can be true, but parsing their relative importance will be a difficult task for future work to unravel (c.f. Passmore et al., 2023; Wood et al., 2022 for initial attempts).

The qualitative analyses raise interesting questions for any generalised conclusion. Does the recurrence of musical motifs represent touchstones of ancient human migrations? Or do humans have an internal preference for particular combinations of sounds and the use of instruments? At least in the case of Berber society, changes in social organisation did not interrupt the evolution of musical style. A quantitative comparison presented some counter-evidence to the Berber conclusion that social organisation did not influence style, with unusual kin organisation encouraging more unusual music in a global comparison, alongside cultural isolation. However, the appearance of unusualness may be affected by the ways regions are classified and by non-linear relationships between cultural diffusion and geographic boundaries (e.g., the rapid expansion of Islamic music and culture throughout North Africa and Western Asia). The practical significance of the social effects also needs to be more thoroughly investigated to draw any definitive conclusion. We do not find strong general support for the proposed hypothesis on isolation and social organisation. However, when it comes to unusual musical styles, a reasonable conclusion may be that the proposed hypotheses could explain unusualness in a particular instance, but not in most instances.

As far as we are aware, Panteli et al. (2017) is the only other study that aims to quantify musical outliers. At the global, and geographically controlled level Panteli et al. point to African and South American countries as musical outliers, whereas our set of outliers are more geographically dispersed. There are two reasons to prefer our approach over Panteli et al. First, as we mentioned in the introduction, is that MIR approaches to musical similarity are yet to align with the musicological experience of similarity which makes the results more difficult to interpret (Daikoku et al., 2022; Ozaki et al., 2021). Although Panteli et al attempt to distil their analytical features into interpretable musical dimensions, evidence suggests these approaches do not currently align with human experience. Another important but equally critical difference is the unit of analysis. Where the Global Jukebox attributes songs to ethnolinguistic groups, Panteli et al assign songs to nation-states. The geographical divisions created by modern nation-states are poor representations of the categorisation of traditional music, which is more reliably linked to the groups which perform them. In Botswana (the country with the most outliers in Panteli et al.), there are 37 distinct languages and dialects, each of which we can assume has distinct musical traditions.

By using human-coded musical features assigned to cultural units, we can be more confident that our measure of unusualness identifies songs that do not fit within their musical region. However, human-coded data also has its own limitations (e.g., subjectivity, reliance on the possibly-biased musical exposure of the listener). While Cantometrics data has been showed to have higher inter-rater reliability and rely less on Western-centric staff notation than most existing global datasets (Wood et al., 2022), it remains imperfect. Indeed, a perfectly objective and unbiased dataset is probably impossible, whether based on human or algorithmic bases. Combining both human and automated methods of analysis (e.g., Mehr et al., 2019; Ozaki et al., Accepted) may prove useful in future studies.

Using the Cantometrics dataset, we identified that unusual songs tend to come from societies with many unusual songs, which we conclude is indicative of musical style. The Cantometric dataset is unevenly sampled, which should be considered alongside the conclusions we draw from our analyses. The unevenness in sampling may have some influence on the calculation of unusualness. Equally, the pattern of sampling within society (i.e. sampling some musical genres more than others), would influence the proportions of state probabilities. A secondary consideration is the impact of geographical regions, as mentioned in the discussion of Moroccan and Berber music. Carving the world into cultural regions is a process fraught with difficulties, and the divisions used as part of the Cantometrics are particularly crude. Changing the scope of geographical regions is likely to impact the scores of particular cases (e.g. which is the most unusual song, or most unusual society), but it is unlikely to affect the more general result, that unusual songs come from societies with unusual music, because these songs are always going to be grouped together. We maintain that the general approach of using quantitative approaches to identify areas of qualitative interest is useful regardless of the decisions made. Future research might investigate how varying the definitions of regions influences the measurement of outliers. Here, we have used statistical approaches, like random effects, to try and mitigate the influence of uneven sampling, however, the gold standard in the future will be to build databases that minimise the influence of bias (Watts et al. 2022).

Songs from the same societies predicting the unusualness of other songs from the same society may seem intuitive, but it is not a foregone conclusion. Recent cross-cultural work in music has routinely emphasised cross-cultural musical *similarities* (Jacoby et al., Accepted; Mehr et al., 2019; Savage et al., 2015; Yurdum et al., In press). This result hopes to encourage further investigation into the patterns in cross-cultural musical *differences*. The Cantometrics dataset is a particularly useful resource for the analyses of cross-cultural differences, and we hope its availability will lead researchers to further investigate the role music plays in human history, its relationships to social organisation, and the possibility of convergent evolution in musical style.

Lomax defined style as “the whole musical communication situation, in which an audience as well as the performers use and understand a common symbolic language” (Lomax, 1980, p. 29). From a quantitative perspective, style might be more practically defined as the proclivity for a particular combination of Cantometric features, independent of genre. The quantification of musical style is what will allow direct testing of many cultural evolutionary hypotheses of musical diversity. Previous work has attempted to analyse style purely by analysing the modal profiles of songs, and while this approach has revealed some interesting cross-cultural correlations (Lomax, 1968), it can be reasonably critiqued for ignoring the prevalence of within-society diversity (Rzeszutek et al., 2012; Savage, 2018). Our analysis of unusualness is performed at the level of songs, and still shows a strong society-level effect, suggesting that there are society-level processes at work that we can detect while accommodating the variation within societies. But our analysis of the unusualness of traditional folk songs is only one way of measuring musical variation. Other research has shown the importance of factors such as genre, age, gender, class, musical training, language, and many other factors (Lomax, 1968; Mehr et al., 2019; Nettl, 2015; Ozaki et al., Accepted; Shilton et al., Accepted). The next steps in understanding musical evolution could incorporate and compare the effects of style, context, and other factors in cross-cultural musical diversity.

## Conclusion

This work has attempted a synthesis of qualitative and quantitative analyses to explore the outlying songs in a global cross-cultural comparison. While the humanistic and scientific analyses of cross-cultural musical diversity often choose one of these methodological devices, this work begins a journey of integrating qualitative and quantitative exploration of musical diversity. Our quantitative measure of unusualness identified the most unusual songs in the dataset: Panpipe-accompanied songs in Kursk, Russia. This work presents a platform for investigating a central debate in the musicology literature: is the similarity in musical style the result of ancient connection, the biological constraints on music production and perception, or how people are allowably organised? Further investigation is needed on this point.

We also compared the musical styles of two culturally related societies, the Moroccan Berber and Kel Aïr. These societies showed similar musical styles, despite significant differences in social organisation. This is evidence against constraints of social organisation and for common inheritance. However, the influence of religion points to an important new influence on musical performance and style. Finally, the best predictor of a song’s unusualness is knowing which society performed a song, suggesting the musical style of a society plays a significant role in defining a musical repertoire. Both qualitative and quantitative comparisons have value in musical research, raising questions and testing generalisations. We hope that by incorporating both approaches we have presented a refined approach to music research that encourages future collaboration and investigation between disciplines and methodologies.

## Data and Methods

### Data

#### Cantometrics

Cantomerics is a dataset of 5,776 songs from 1,026 societies, manually coded on 37 different variables (Wood et al. 2022). Most original recordings can be listened to at www.theglobaljukebox.org (with exceptions due to copyright). Data was largely collected between the 1940s to 1980s, with a maximum range of 1904 to 1982. Cantometric variables were developed through the field observation, listening, and experimentation of creators Alan Lomax and Victor Grauer. They cover topics such as: the social organizational, integrative, and differentiating aspects of performance; vocal qualities, ornament, and articulation; melodic and rhythmic characteristics and structure; and the relationship between vocal and orchestral parts. Only variables that could be reliably distinguished were included. The goal of Cantometrics was to develop a coding scheme that would capture musical diversity and performance, without the reliance and restrictions of Western staff notation.

We use Cantometrics as it was coded by Lomax and Grauer, which can be somewhat unintuitive. Cantometrics values were arranged to reflect their respective contributions to greater “articulation or individualization” on the left end of the scale, to greater integration on the right side. Some variables were somewhat neutral in these respects, and in others, the values were somewhat or quite independent.

#### The Ethnographic Atlas

The Ethnographic Atlas describes cultural practices for 1291 societies, ranging from societies with complex agricultural economies and political systems to small hunter-gatherer groups. The societies are globally distributed with especially good coverage of Africa and western North America. The Ethnographic Atlas data is largely coded by the author, George Murdock, cross-referenced across ethnographic sources, with some codings provided or corrected by field researchers. We use the digitised version of the Ethnographic Atlas stored on D-PLACE (Kirby et al. 2016). Ethnographic Atlas data was largely collected between 1900 and 1950. Two societies were collected later than 1950, and 30 before 1900.

#### Global Phylogeny

We use a global language phylogeny to identify cultural isolation (Bouckaert et al. 2022). The global language phylogeny was built from a taxonomy of extant languages, together with Bayesian phylogenetic analysis of basic vocabulary data, information on language diversification events, geographic locations of languages, and assumptions on the paths of human migration.

### Methods

#### Unusualness

As described in the main text, unusualness is the log-likelihood of a song within a region, excluding the influence of all other songs from that society. Log-likelihoods are calculated by first calculating the proportion of occurrence for each state within each Cantometric feature, for a particular region. State probabilities exclude all songs from the society of interest. Then using these values to calculate the log-likelihood we take the log sum of the probabilities for the relevant state of each Cantometric feature for a song. Regions are a geographical categorisation within Cantometrics that broadly correspond to United Nations Regional Groupings (https://unstats.un.org/sdgs/report/2019/regional-groups/). A concern in the calculation of unusualness by region is that societies with a larger sample will have a greater influence on unusualness. A weak correlation between the average unusualness scores for a society and the number of songs recorded for that society suggests that this bias is unlikely to have an effect (Pearson’s Correlation = 0.04).

#### Bayesian Multi-level regression

##### Choice of distance metric

We have no prior predictions of what geographic distance will best represent the frequency of contact. To decide on a distance, we create three variables with decreasing distances: 1000 km radius from a particular society, 500 km radius, and 250 km radius and ask which variable shows the strongest relationship to unusualness, in a bi-variate (one response variable, one predictor variable) regression, with a random effect for society. All variables are standardized. We compare model fit using Leave-One-Out (LOO) expected log pointwise predictive density (ELPD). LOO comparison involves recalculating the model, with one data point removed each time and calculating the predictive value of that model. Finally, we calculate the ELPD to give an average predictive value of that model. The difference between all models is small (<4; (Sivula et al., 2022), with a minor preference for a 500 km radius, which is used in all future analyses.

##### Model Validation

The final model presented in the paper is in the following form:


\begin{array}{l}
Unusualness \sim N\ Neighbour{s_{500km}} + Cultural\ Distance + Kinship\ unusualness + \\
\,\,\,\,\,\,\,\,\,\,\,\,\,\,\,\,\,\,\,\,\,\,\,\,\,\,\,\,\,\,\,\,\,\,\,Economic\ Unusualness + {Society\ mean_{unusualness}}
\end{array}

All variables are normalized to have a mean of 0 and a standard deviation of 1. The model is run for four chains, for 4,000 iterations and a burn-in of 2,000 iterations. We also ran a 10-fold cross-validation model. 10-fold cross-validation splits the data into 10 chunks and runs the model nine times, removing one chunk each time. This process tells us that our conclusions are not dependent on a particular subset of data. All estimates have an R-hat of 1, and the effective sample size is similar to the number of model iterations, indicating chain efficiency. LOO comparison shows that this model is considerably better than an intercept-only model (ELPD = –444) but not better than a model only containing the mean unusualness value for a society.

## Data Accessibility Statement

All data and analysis code are publicly available at https://github.com/SamPassmore/musical_unusualness. See Wood et al. (2022) for a detailed description of the full Cantometric dataset (https://github.com/theglobaljukebox/cantometrics), including streaming audio recordings and a detailed explanation of the Cantometric coding scheme with audio examples (http://theglobaljukebox.org). Please cite Wood et. al (2022) if using Cantometrics, or other Global Jukebox data.

## Footnotes

You can listen to these songs using their song identifiers at www.theglobaljukebox.org.
